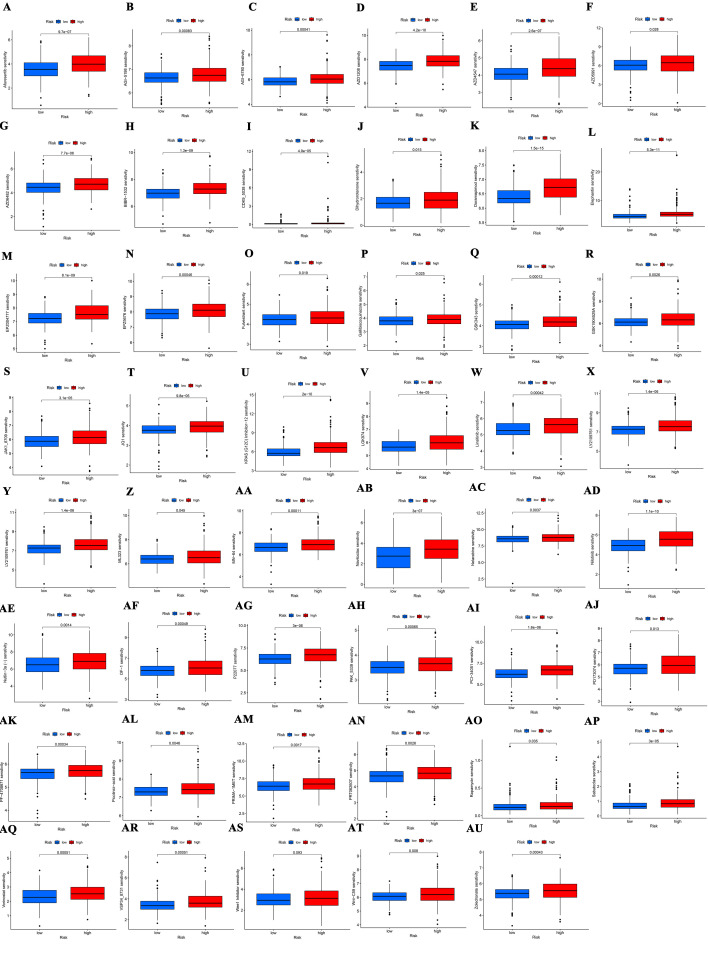# Construction and validation of a prognostic model for bladder cancer based on disulfidptosis-related lncRNAs

**DOI:** 10.1097/MD.0000000000038750

**Published:** 2024-07-05

**Authors:** Xiaoyu Yang, Yunzhi Zhang, Jun Liu, Yougang Feng

**Affiliations:** a Department of Urology, Suining Central Hospital, Suining, Sichuan, China; b Department of Gastroenterology, Suining Central Hospital, Suining, Sichuan, China.

**Keywords:** bladder cancer, disulfidptosis, long non-coding RNAs, risk prognostic model

## Abstract

**Background:**

Bladder cancer (BLCA) is a prevalent and aggressive cancer associated with high mortality and poor prognosis. Currently, studies on the role of disulfidptosis-related long non-coding RNAs (DRLs) in BLCA are limited. This study aims to construct a prognostic model based on DRLs to improve the accuracy of survival predictions for patients and identify novel targets for therapeutic intervention in BLCA management.

**Methods:**

Transcriptomic and clinical datasets for patients with BLCA were obtained from The Cancer Genome Atlas. Using multivariate Cox regression and least absolute shrinkage and selection operator techniques, a risk prognostic signature defined by DRLs was developed. The model’s accuracy and prognostic relevance were assessed through Kaplan–Meier survival plots, receiver operating characteristic curves, concordance index, and principal component analysis. Functional and pathway enrichment analyses, including Gene Ontology, Kyoto Encyclopedia of Genes and Genomes, and Gene Set Enrichment Analysis, were conducted to elucidate the underlying biological processes. Immune cell infiltration was quantified using the CIBERSORT algorithm. Differences and functions of immune cells in different risk groups were evaluated through single-sample Gene Set Enrichment Analysis. The Tumor Immune Dysfunction and Exclusion predictor and tumor mutational burden (TMB) assessments were utilized to gauge the likelihood of response to immunotherapy. Drug sensitivity predictions were made using the Genomics of Drug Sensitivity in Cancer database.

**Results:**

A robust 8-DRL risk prognostic model, comprising LINC00513, SMARCA5-AS1, MIR4435-2HG, MIR4713HG, AL122035.1, AL359762.3, AC006160.1, and AL590428.1, was identified as an independent prognostic indicator. This model demonstrated strong predictive power for overall survival in patients with BLCA, revealing significant disparities between high- and low-risk groups regarding tumor microenvironment, immune infiltration, immune functions, TMB, Tumor Immune Dysfunction and Exclusion scores, and drug susceptibility.

**Conclusion:**

This study introduces an innovative prognostic signature of 8 DRLs, offering a valuable prognostic tool and potential therapeutic targets for bladder carcinoma. The findings have significant implications for TMB, the immune landscape, and patient responsiveness to immunotherapy and targeted treatments.

## 1. Introduction

Bladder cancer (BLCA) presents a critical health issue, predominantly impacting middle-aged and elderly populations.^[1]^ Projections indicate it will rank as the fourth most common and eighth deadliest cancer among men by 2024.^[2]^ Despite the efficacy of traditional treatments, such as transurethral resection of bladder tumor, radical cystectomy, and chemotherapy, the recurrence and progression to metastasis remain significant challenges.^[3,4]^ Recent years have witnessed substantial advancements in systemic treatment options for advanced BLCA.^[5]^ Notably, immunotherapy advancements, particularly PD-1/PD-L1 inhibitors, have shown promise in treating advanced cases.^[6]^ Immune checkpoint inhibitors have yielded notable clinical responses and survival benefits in patients with advanced and refractory BLCA.^[7,8]^ However, overall response rates remain relatively low,^[9]^ and adverse reactions associated with immunotherapy, coupled with patients’ nutritional status, further constrain its application.^[10,11]^ The mechanisms of immune evasion in BLCA are increasingly being elucidated, offering insights into the variable responses to immunotherapy.^[12]^ Furthermore, emerging therapies, including targeted therapies and antibody-drug conjugates, are being investigated, offering new hope for improved patient outcomes.^[13]^ Despite these advancements, there is an urgent need to identify novel biomarkers and therapeutic targets to enhance treatment efficacy and patient prognosis.^[14]^

Disulfidptosis is a recently discovered form of programmed cell death that distinguishes itself from traditional mechanisms such as apoptosis,^[15]^ autophagy,^[16]^ and necroptosis.^[17]^ This process is initiated by a deficiency in NADPH during cancer cell starvation, which disrupts cysteine metabolism through SLC7A11, leading to the accumulation of abnormal disulfide bonds. This accumulation induces disulfide stress that impairs actin proteins within the cell’s cytoskeleton, ultimately resulting in cell death.^[18]^ Exploiting the metabolic vulnerability associated with SLC7A11 and its related pathways to induce disulfidptosis offers promising targets for the development of innovative cancer treatment strategies.^[19]^

Long non-coding RNAs (lncRNAs) are a diverse group of RNA molecules that, despite not encoding proteins, play pivotal roles in various cellular functions.^[20]^ LncRNAs are increasingly recognized for their involvement in the development and progression of BLCA.^[21]^ For instance, the lncRNA RP11-89 act as a competitive endogenous RNA for miR-129-5p in BLCA, facilitating tumorigenesis and resistance to ferroptosis via the upregulation of PROM2.^[22]^ Another lncRNA, IGF2BP2-AS1, has been implicated in immune evasion and modulation of the tumor microenvironment (TME).^[23]^ A comprehensive understanding of the functional roles and regulatory mechanisms of lncRNAs could elucidate the pathogenesis of BLCA and pave the way for novel diagnostic and therapeutic approaches.^[24]^ The predictive value of disulfidptosis-related long non-coding RNAs (DRLs) and their correlation with the immune landscape in BLCA remains underexplored. Therefore, this study aims to develop a novel DRL signature to predict prognosis and immune response.

## 2. Materials and methods

### 2.1. Data acquisition

RNA sequencing transcriptome data, clinical information, and somatic mutation data for 19 standard and 409 BLCA samples were retrieved from The Cancer Genome Atlas (TCGA) database. Standard control samples were excluded, and cases with insufficient survival time, age, and tumor stage information were eliminated. Furthermore, genes related disulfidptosis (GYS1, NDUFS1, OXSM, LRPPRC, NDUFA11, NUBPL, NCKAP1, RPN1, SLC3A2, SLC7A11) were sourced from pertinent published studies.^[25,26]^

### 2.2. LncRNA screening and establishment of the model

A total of 409 BLCA specimens were randomly divided into training and test cohorts using the “caret” package. To identify lncRNAs associated with disulfidptosis, Pearson correlation analysis was performed, focusing on lncRNAs that exhibited co-expression with disulfidptosis-related genes. A stringent selection criterion was applied, considering, only lncRNAs with an absolute correlation coefficient >0.4 and a *P* value <.001 as disulfidptosis-related. These selected lncRNAs were then depicted using a Sankey diagram for enhanced visualization. In the training cohort, univariate Cox regression analysis was applied to filter for lncRNAs significantly impacting overall survival (OS), and the results were presented in a comprehensive forest plot. Subsequent refinement using the least absolute shrinkage and selection operator and multivariate Cox regression analyses isolated the most impactful subset of OS-related lncRNAs, which were used to develop a prognostic signature. This involved calculating individual risk scores by multiplying each lncRNA’s expression level by its corresponding regression coefficient.

### 2.3. Validation of the model

The precision of the risk model was evaluated using the concordance index (C-index) and receiver operating characteristic (ROC) curves. Additionally, Kaplan–Meier survival analysis was employed to assess the prognostic independence of the risk score across the stratified risk groups. The risk score’s status as an independent prognostic indicator was further validated through both univariate and multivariate Cox regression analyses. To enhance clinical applicability, a comprehensive nomogram integrating the risk score with patient clinical features was developed to predict OS, with its predictive performance assessed using a calibration curve. Principal component analysis (PCA) and risk score correlation assessments conducted to confirm the discriminatory power of the risk model between high-risk and low-risk patient groups.

### 2.4. Function and pathway analyses

Differentially expressed genes (DEGs) between the 2 risk cohorts were identified using the “limma” package. Subsequently, functional enrichment analysis of these DEGs was performed using the “clusterProfiler,” “org.HS.e.g..db,” and ‘enrichplot’ packages to analyze Gene Ontology (GO) terms and Kyoto Encyclopedia of Genes and Genomes (KEGG) pathways. The DOSE package was employed to perform Gene Set Enrichment Analysis (GSEA) to examine the enrichment of KEGG pathways in high-risk and low-risk groups. The criteria for significance were set as an absolute logFC >1, false discovery rate <0.25, and a *P* value <.05.

### 2.5. Immune infiltration and functional analysis

The “estimate” package was implemented to assess the TME by calculating scores indicative of stromal and immune cell presence. To further delineate the differences in stromal and immune cell composition, and their aggregate scores between high- and low-risk cohorts, the “reshape2” and “ggpubr” packages were employed. Additionally, the CIBERSORT algorithm to determine the relative abundance of various immune cell types within each sample. The analysis was expanded using single-sample GSEA, enabling a comparative investigation of the variations in immune cell populations and functional immune scores across risk stratifications. To evaluate the likelihood of response to immunotherapy, the Tumor Immune Dysfunction and Exclusion (TIDE) platform was used, predicting TIDE scores to identify differences in immunotherapeutic outcomes between high- and low-risk groups.

### 2.6. Tumor mutational burden (TMB) analysis

Using the Perl programming language, the mutational burden and frequency within BLCA genomic data sourced from TCGA were evaluated. The top 15 most frequently mutated genes were selected to elucidate the variation in gene mutation rates between the high-risk and low-risk groups. These genes were graphically represented in a waterfall plot using the “maftools” R package, offering a clear visual comparison. The “limma” package in R was utilized to analyze and quantify the differences in TMB between the 2 cohorts. Furthermore, Kaplan–Meier survival analysis was performed to investigate the relationship between TMB – both as an independent metric and in combination with the risk score – and patient survival outcomes in BLCA.

### 2.7. Drug sensitivity prediction

To predict the drug sensitivity profiles of patients with BLCA, the “oncoPredict” R package, which utilizes training data from the Genomics of Drug Sensitivity in Cancer database, was employed. The “calcPhenotype” function within the package was used to generate individual drug sensitivity scores for each patient. These scores enabled the identification of pharmacological agents exhibiting differential effectiveness between patients classified within into high- and low-risk groups.

### 2.8. Statistical analysis

Data manipulation and analysis were executed using Strawberry Perl (version 5.30.0) and R software (version 4.3.1). Graphical representations and statistical assessments were produced with R software (version 4.2.2). Continuous variable comparisons between 2 groups employed both parametric and non-parametric tests, whereas categorical data comparisons utilized the chi-square test. Statistical significance was established at *P* value threshold of <.05.

## 3. Results

### 3.1. Identification of disulfdptosis-related lncRNAs and establishment of a predictive model for BLCA

**Figure 1.**
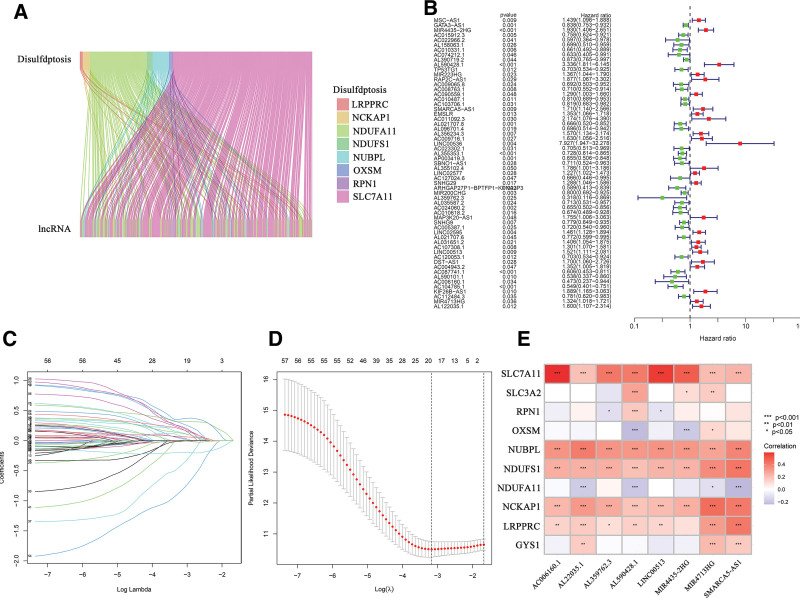
Development of a prognostic signature based on disulfidptosis-related lncRNAs. (A) Sankey diagram illustrating the association between lncRNAs and disulfidptosis-related genes. (B) Forest plot representing the prognostic significance of disulfidptosis-related lncRNAs. (C) Diagram illustrating LASSO expression coefficients of 59 disulfidptosis-related lncRNAs (D) Cross-validation curve of LASSO regression. (E) Heatmap depicting the correlation between model-included lncRNAs and disulfidptosis-related genes (significance levels denoted as *P < .05, **P < .01, ***P < .001). LASSO = least absolute shrinkage and selection operator.

Patients with BLCA were randomly allocated into a training set and a test set. The clinical characteristics of the participants are presented in Table 1. Pearson correlation analysis identified 326 DRLs, depicted in Figure 1A, and detailed in Supplementary Table S1, Supplemental Digital Content, http://links.lww.com/MD/N93. Subsequently, within the training set, 59 prognostically significant DRLs were isolated through univariate analysis, as shown in Figure 1B. A prognostic model consisting of 8 DRLs was then refined using least absolute shrinkage and selection operator and multivariate Cox regression analyses, illustrated in Figure 1C and D, respectively. The risk score for each patient was calculated based on this model using the following equation: risk score = (0.389 × expression value of MIR4435-2HG) + (0.543 × expression value of SMARCA5-AS1) − (1.357 × expression value of AL359762.3) + (1.002 × expression value of LINC00513) − (1.464 × expression value of AC006160.1) + (0.430 × expression value of MIR4713HG) + (0.351 × expression level of AL122035.1) − (0.587 × expression value of AL590428.1). Furthermore, Figure 1E provides heat maps illustrating the expression correlations between these 8 pivotal lncRNAs and disulfidptosis-associated genes.

**Table 1:** Clinicopathological characteristics of patients with BLCA.

Covariates	Type	Total	Test	Train	*P* value
Age	≤65	158 (39.7%)	79 (39.7%)	79 (39.7%)	1
	>65	240 (60.3%)	120 (60.3%)	120 (60.3%)	
Gender	Female	102 (25.63%)	49 (24.62%)	53 (26.63%)	.7305
	Male	296 (74.37%)	150 (75.38%)	146 (73.37%)	
Grade	High grade	375 (94.22%)	183 (91.96%)	192 (96.48%)	.1056
	Low grade	20 (5.03%)	14 (7.04%)	6 (3.02%)	
	Unknown	3 (0.75%)	2 (1.01%)	1 (0.5%)	
Stage	Stage I	2 (0.5%)	0 (0%)	2 (1.01%)	.436
	Stage II	126 (31.66%)	65 (32.66%)	61 (30.65%)	
	Stage III	138 (34.67%)	71 (35.68%)	67 (33.67%)	
	Stage IV	130 (32.66%)	61 (30.65%)	69 (34.67%)	
	Unknown	2 (0.5%)	2 (1.01%)	0 (0%)	
T	T0	1 (0.25%)	0 (0%)	1 (0.5%)	.759
	T1	3 (0.75%)	1 (0.5%)	2 (1.01%)	
	T2	116 (29.15%)	58 (29.15%)	58 (29.15%)	
	T3	190 (47.74%)	96 (48.24%)	94 (47.24%)	
	T4	56 (14.07%)	26 (13.07%)	30 (15.08%)	
	TX	1 (0.25%)	1 (0.5%)	0 (0%)	
	Unknown	31 (7.79%)	17 (8.54%)	14 (7.04%)	
M	M0	192 (48.24%)	101 (50.75%)	91 (45.73%)	1
	M1	11 (2.76%)	6 (3.02%)	5 (2.51%)	
	Unknown	195 (48.99%)	92 (46.23%)	103 (51.76%)	
N	N0	232 (58.29%)	123 (61.81%)	109 (54.77%)	.5119
	N1	44 (11.06%)	24 (12.06%)	20 (10.05%)	
	N2	75 (18.84%)	34 (17.09%)	41 (20.6%)	
	N3	6 (1.51%)	2 (1.01%)	4 (2.01%)	
	Unknown	41 (10.3%)	16 (8.04%)	25 (12.56%)	

BLCA = bladder cancer.

### 3.2. Assessment and validation of the disulfidptosis-related lncRNAs model for BLCA

**Figure 2.**
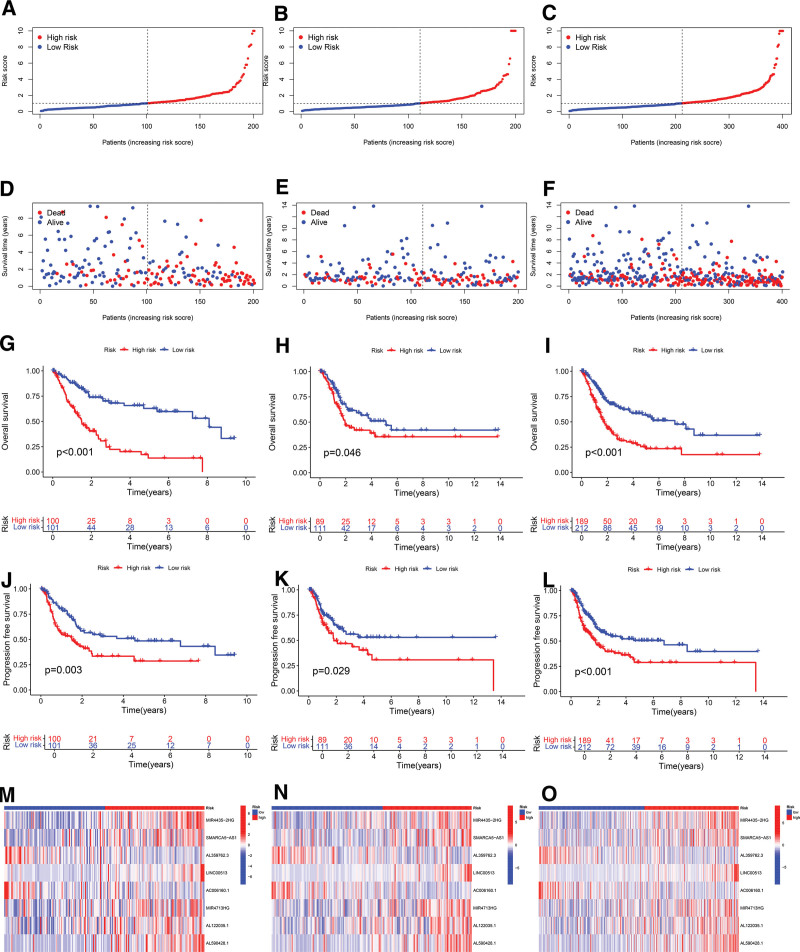
Assessment and validation the prognostic value of the DRLs signature. (A–C) Risk score distribution charts in the training, test, and entire sets. (D–F) Distribution of survival outcomes for patients with BLCA across risk groups in the training, test, and entire sets, respectively. (G–I) Kaplan–Meier survival plots for overall patient survival across the training, test and entire sets, respectively. (J–L) Kaplan–Meier estimates for progression-free survival of patients in the training, test, and entire sets, respectively. (M–O) Expression profiles of 8 DRLs displayed in heatmaps for samples across high- and low-risk groups in the training, test, and entire sets respectively. BLCA = bladder cancer, DRLs = disulfidptosis-related long non-coding RNAs.

Patients from the training, testing, and entire cohorts were stratified based on their risk scores. Utilizing the median risk score from the training cohort as a benchmark, individuals in each cohort were classified into high-risk and low-risk groups (Fig. 2A–C). A significant correlation was observed, indicating that higher risk scores were associated with increased mortality rates (Fig. 2D–F). Survival analyses demonstrated that high-risk patients exhibited significantly poorer OS compared to those in the low-risk group across the training cohort, a trend consistently observed in both the testing and entire datasets (Fig. 2G–I). Additionally, Figure 2J to L illustrates that individuals categorized as low-risk generally experienced superior progression-free survival outcomes compared to those designated as high-risk. Risk heatmap analysis, as shown in Figure 2M to O, revealed that expression levels of the lncRNAs MIR4435-2HG, SMARCA5-AS1, LINC00513, MIR4713HG, AL590428.1, and AL122035.1 were significantly higher in high-risk patients compared to those in the low-risk group, a pattern consistent across all 3 cohorts.

**Figure 3.**
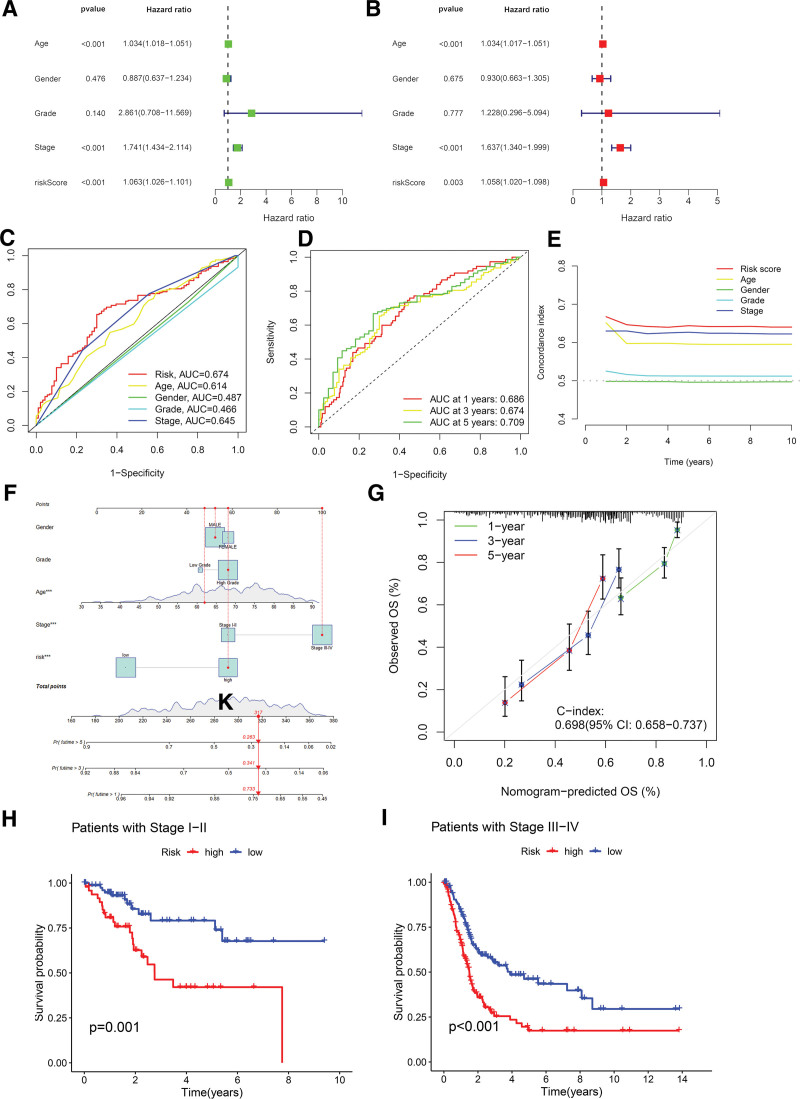
Validation and prognostic assessment of the disulfdptosis-related lncRNA signature as an independent risk factor. (A) Univariate COX regression analysis of clinical pathological features and risk scores. (B) Multivariate COX regression analysis of clinical pathological features and risk scores. (C) Predictive accuracy of the risk scoring model compared with clinical pathological features based on the AUC values of the ROC curves. (D) Predictive accuracy of the risk scoring model features based on the AUC values of the ROC curves at 1, 3, and 5 years. (E) Concordance index curve of the risk factors, including risk score, age, gender, and tumor grade. (F) Nomogram incorporating risk score and clinicopathological factors to predict 1-, 3-, and 5-year OS in patients with BLCA. (G) Calibration curves showing the accuracy of risk prediction models in predicting 1-, 3-, and 5-year OS in patients with BLCA compared with actual 1-, 3-, and 5-year OS in patients with BLCA. (H–I) Kaplan–Meier survival plots illustrating the overall survival differences between high- and low-risk patients with BLCA in early and late stages of the disease. BLCA = bladder cancer, ROC = receiver operating characteristic, OS = overall survival.

To evaluate the risk score’s potential as an independent prognostic factor and its predictive performance, univariate regression, multivariate regression, ROC curve, and C-index analyses were conducted. The univariate and multivariate Cox regression analyses identified age, stage, and risk score as independent prognostic markers for patients with BLCA (Fig. 3A and B). By integrating the risk score with additional clinical features, a ROC curve was constructed, demonstrating that the area under the ROC curve (AUC) for the risk score surpassed those of other clinical characteristics (Fig. 3C). Specifically, the risk score’s AUC at 1, 3, and 5 years were 0.686, 0.674, and 0.709, respectively (Fig. 3D). Furthermore, the 10-year C-index for the risk model exceeded those of other clinical model variables (Fig. 3E), highlighting the prognostic model’s independence and superior predictive accuracy compared to other clinical factors.

**Figure 4.**
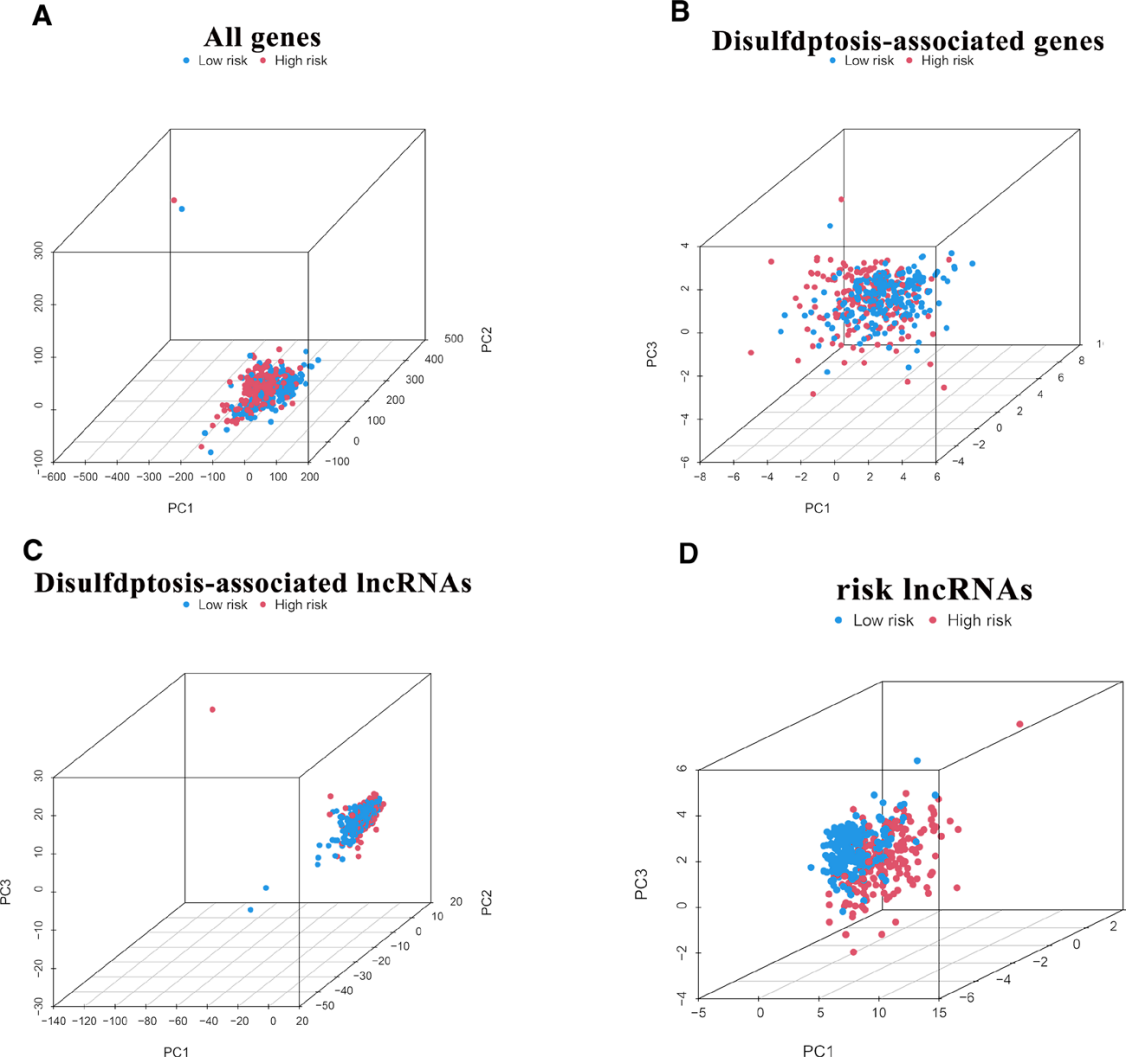
Principal component analysis (PCA). (A) PCA of all gene expression. (B) PCA of disulfdptosis-related genes. (C) PCA of disulfdptosis-related lncRNAs. (D) PCA of model risk-related lncRNAs.

To enhance the precision of patient outcome predictions, a nomogram was developed that integrates clinicopathological factors with the risk score, predicting 1-year, 3-year, and 5-year OS probabilities of 0.789, 0.442, and 0.361, respectively (Fig. 3F). Calibration curves indicated a strong correlation between nomogram-predicted survival probabilities and actual observed survival rates at the 1-, 3-, and 5-year benchmarks (Fig. 3G). Additionally, the model’s predictive superiority over tumor stage alone was evaluated by stratifying patients with BLCA into early-stage (stage I or II) and advanced-stage (stage III or IV) groups. This stratification revealed that, within each stage group, high-risk patients consistently exhibited significantly lower OS rates compared to low-risk patients (Fig. 3H and I), highlighting the risk score’s prognostic value across various disease stages. Furthermore, PCA was performed on several models, including those based on all genes, disulfidptosis-associated genes, disulfidptosis-associated lncRNAs, and the risk lncRNAs model, to evaluate their ability to distinguish between high-risk and low-risk patients with BLCA. The results demonstrated that our constructed model effectively separated the 2 risk groups, emphasizing its precision (Fig. 4A and D). These findings suggest that risk scores could serve as a reliable prognostic marker for predicting survival rates in patients with BLCA.

### 3.3. Functional and pathway analysis

**Figure 5.**
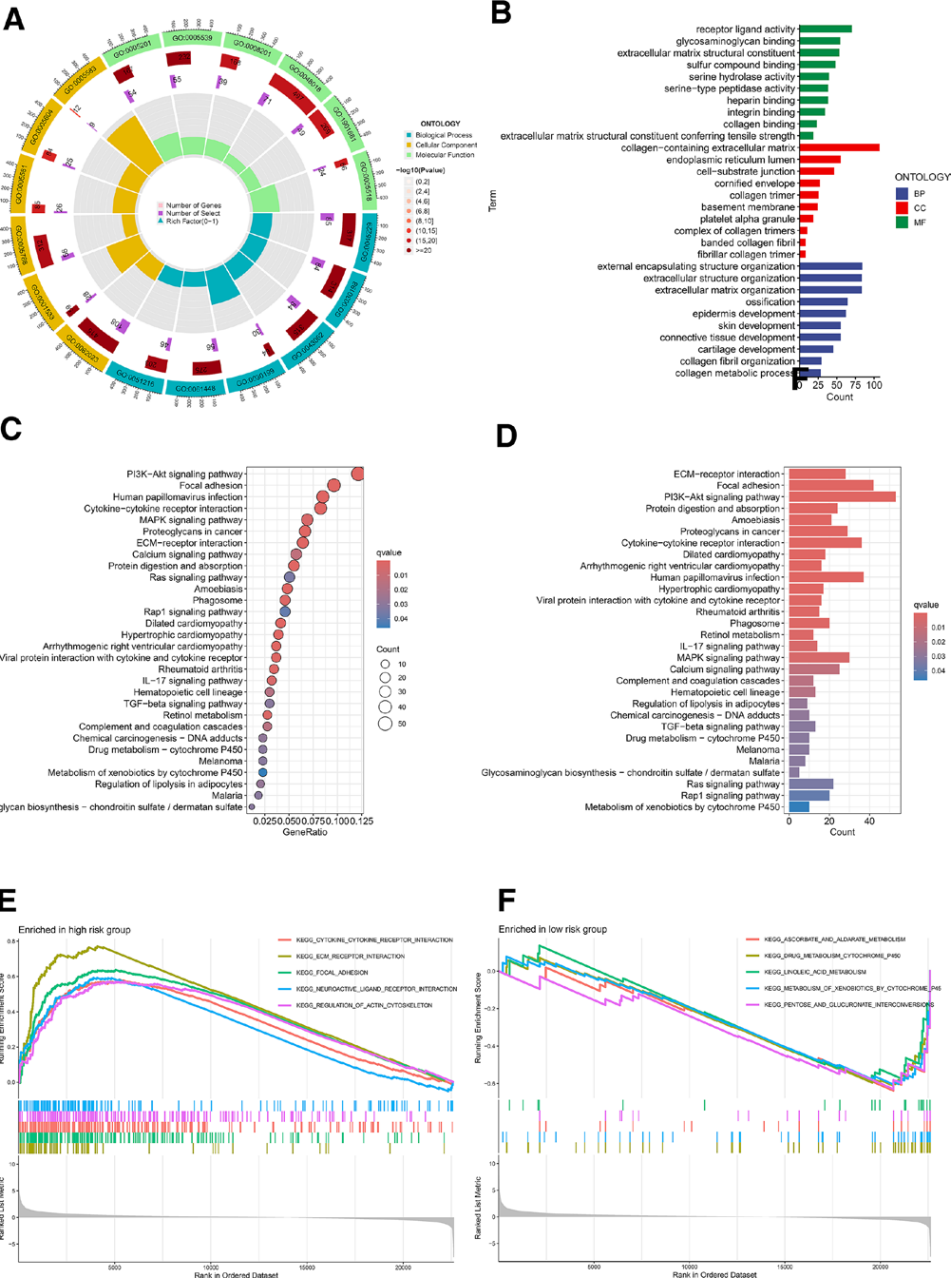
GO, KEGG, and GSEA enrichment analyses. (A) Circular graph showing GO analysis results. (B) Bar chart displaying GO analysis results. (C) Bubble chart displays KEGG pathway analysis results. (D) Bar chart displays KEGG pathway analysis results. (E) GSEA pathway enrichment analysis for the high-risk group. (F) GSEA pathway enrichment analysis for the low-risk group. GO = Gene Ontology, GSEA = Gene Set Enrichment Analysis, KEGG = Kyoto Encyclopedia of Genes and Genomes.

To explore the biological characteristics of genes associated with high-risk and low-risk groups, GO and KEGG pathway analyses were conducted. A total of 1199 DEGs were identified between the 2 groups. GO analysis revealed biological process enrichments related to the assembly of external encapsulating structures, extracellular structures, and extracellular matrix (ECM). Cellular components were predominantly associated with the collagen-containing ECM, the endoplasmic reticulum lumen, and cell-substrate junctions. Enriched molecular functions included receptor-ligand activity, glycosaminoglycan binding, and structural constituents of the ECM (Fig. 5A and B). KEGG pathway analysis indicated an overrepresentation of pathways such as the phosphatidylinositol-3-kinase-Akt (PI3K-Akt) signaling pathway, focal adhesion, and ECM-receptor interactions (Fig. 5C and D). Furthermore, GSEA revealed that pathways related to focal adhesion, cytokine-cytokine receptor interaction, and ECM-receptor interaction were predominant in the high-risk group (Fig. 5E). In contrast, pathways related to ascorbic acid and malonate metabolism, cytochrome P450-dependent drug metabolism, and linoleic acid metabolism were more active in the low-risk group (Fig. 5F).

### 3.4. TME analysis and immunotherapy outcomes

**Figure 6.**
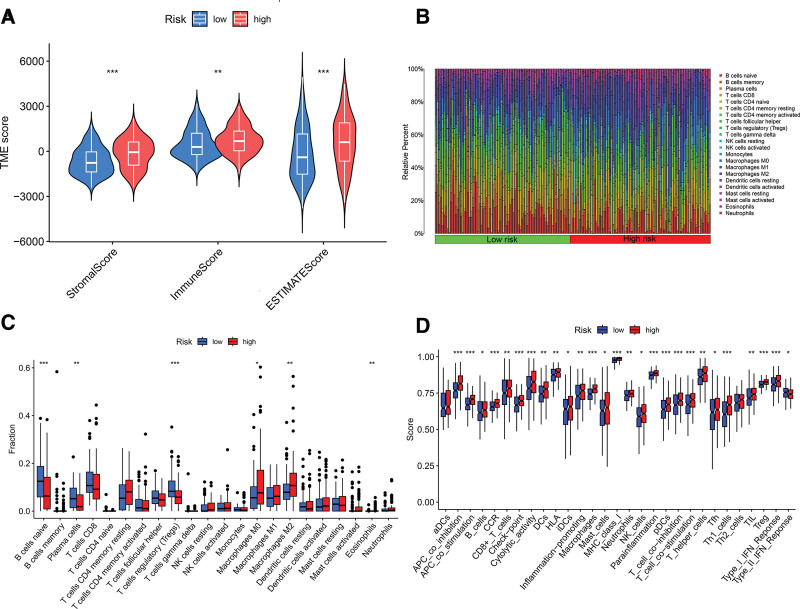
Analysis of the tumor immune microenvironment. (A) Stromal score, Immune score, and ESTIMATE score analyses for high- and low-risk groups. (B) Immune cell percentage for high- and low-risk groups. (C) Immune cell infiltration for high- and low-risk groups. (D) Immunofunctional analysis for high- and low-risk groups. (*P < .05; **P < .01; ***P < .001).

The tumor immune microenvironment is critical for the effectiveness of immunotherapy. Recent research has shown that the ECM, a non-cellular component of the TME, can regulate immune cells, creating an immunosuppressive environment that hinders the success of immunotherapeutic treatments.^[27,28]^ Analysis revealed significant ECM enrichment in the high-risk group, suggesting potential differences in the immune microenvironment between high- and low-risk BLCA groups. Utilizing the ESTIMATE algorithm, both stromal and immune scores were significantly elevated in the high-risk group, indicating heightened immune cell infiltration (Fig. 6A). To further dissect the immune cell landscape, the CIBERSORT algorithm was applied, quantifying the proportional representation of various immune cell types within each patient sample. Figure 6B illustrates the comparative distribution and disparities of 22 immune cell populations between the high- and low-risk BLCA patient groups. Additionally, single-sample GSEA was used to delineate variances in immune cell types and immune function scores between the 2 risk groups. The analysis indicated a greater presence of M0 and M2 macrophages, alongside a decrease in T regulatory cell infiltration in the high-risk BLCA group (Fig. 6C). Furthermore, the high-risk BLCA subset demonstrated elevated function scores across 27 of the 29 assessed immune functions compared to their low-risk counterparts (Fig. 6D).

### 3.5. TMB and TIDE analyses of the signature

**Figure 7.**
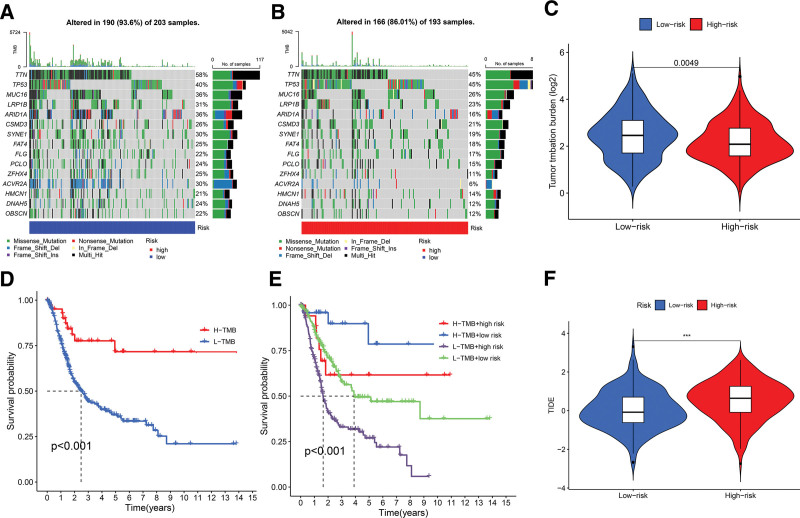
TMB and TIDE analyses. (A) Waterfall plot illustrating somatic mutations in tumors classified with a low-risk score. (B) Waterfall plot illustrating somatic mutations in tumors classified with a high-risk score. (C) Comparison of TMB levels between low- and high-risk groups. (D) Kaplan–Meier survival curves demonstrating the impact of TMB classification on overall survival (OS). (E) Kaplan–Meier survival analysis for OS based on a combination of TMB classification and risk assessment. (F) Comparison of TIDE scores between high- and low-risk groups. (***P < .001). TIDE = tumor immune dysfunction and exclusion, TMB = tumor mutational burden.

TMB quantifies the number of somatic mutations per megabase of the genomic sequence. In this study, the mutation frequency in nearly all studied genes was higher in the low-risk group compared to the high-risk group. The 5 most frequently mutated genes in the analyzed samples were TP53, TTN, MUC16, LRP1B, and ARID1A (Fig. 7A and B). Additionally, a comparative analysis of TMB and gene mutation frequencies was performed across low-risk and high-risk BLCA groups. Findings revealed that the low-risk BLCA group exhibited a significantly elevated TMB (Fig. 7C).

Notably, a high TMB correlated with better OS; patients with a high TMB had significantly better survival rates than those with a low TMB (Fig. 7D). The combined effects of risk score and TMB on survival were also explored by stratifying patients into 4 distinct groups. The subgroup with a high TMB and low-risk score demonstrated the best prognosis, with an impressive 10-year survival rate of approximately 75%. Conversely, patients with a low TMB and high-risk score had the worst prognosis, with a 5-year survival rate of about 25%. Interestingly, there was no significant difference in survival rates between high-risk patients with a high TMB and low-risk patients with a low TMB (Fig. 7E). These results underscore the potential of TMB as a valuable predictive biomarker for survival outcomes in patients with BLCA.

Additionally, the TIDE methodology was utilized to assess the likelihood of tumor immune evasion in patients. Analysis indicated significantly higher TIDE scores in the high-risk group, suggesting an increased tendency for immune escape and potentially reduced effectiveness of immunotherapy in these patients (Fig. 7F). These insights may provide critical guidance for tailoring immunotherapeutic strategies in BLCA.

### 3.6. Potential therapeutic agents in BLCA

**Figure 8.**
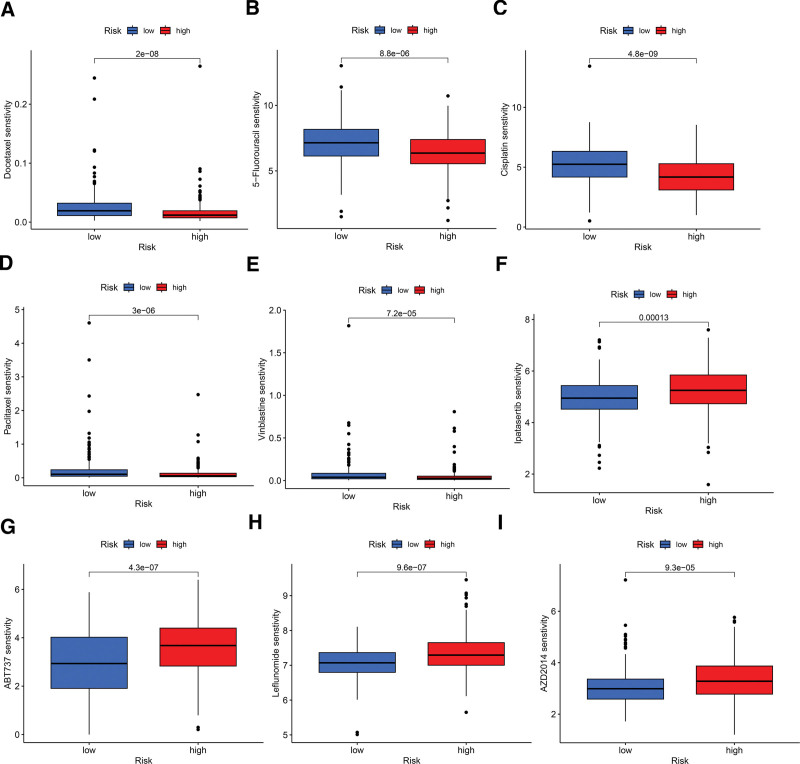
Sensitivity of patients with BLCA in both groups to various drugs. (A) Docetaxel. (B) 5-Fluorouracil. (C) Cisplatin. (D) paclitaxel. (E) Vinorelbine. (F) Ipatasertib. (G) ABT-737. (H) Leflunomide. (I) AZD20143.7. BLCA = bladder cancer.

In this study, the relationship between BLCA risk scores and sensitivity to various anticancer drugs was also examined. Utilizing the “calcPhenotype” function in the “oncoPredict” package, sensitivity scores were calculated. Results indicated that 68 drugs exhibited increased sensitivity in the high-risk group, while 56 drugs were more sensitive in the low-risk group. Figure 8A to I display the sensitivity profiles for selected anticancer drugs that are either commonly used or identified as potential treatments for patients with BLCA. Sensitivity data for other drugs assessed in both risk groups are detailed in Supplementary Figure S1, Supplemental Digital Content, http://links.lww.com/MD/N91 and Supplementary Figure S2, Supplemental Digital Content, http://links.lww.com/MD/N92.

## 4. Discussion

Cancer cells often exist in a microenvironment characterized by elevated stress levels, limited nutrients, and hypoxia.^[29]^ This hostile environment poses significant challenges to the survival and growth of cancer cells.^[30]^ Cysteine is crucial for protecting cells from oxidative damage and maintaining protein structure stability by regulating disulfide bond formation of disulfide bonds.^[15,31]^ However, cancer cells cannot obtain sufficient cysteine to meet their antioxidant needs through endogenous synthesis or protein degradation.^[32]^ Consequently, cancer cells upregulate the expression of the cystine transporter protein (SLC7A11) through metabolic reprogramming to acquire extracellular cystine, which is then reduced to cysteine in the cytoplasm to maintain redox homeostasis.^[33]^ Gan and colleagues have demonstrated that when cancer cells are starved, inhibition of the pentose phosphate pathway leads to a lack of NADPH production, resulting in the accumulation of cystine in the cytoplasm and the formation of excessive harmful disulfide bonds.^[34]^ The cytoplasmic system that reduces disulfide bonds, dependent on NADPH, becomes overwhelmed and unable to clear these harmful bonds, causing an accumulation that induces disulfide bond stress.^[35]^ This stress activates the Rac1-WRC-Arp2/3 signaling pathway, disrupting the interaction of cytoskeletal proteins and ultimately compromising cell structural integrity, leading to cell death.^[34,36]^ Reversing cell death and oxidative defects caused by disulfide bond stress can be achieved by using reducing agents or supplementing NADPH.^[37]^ The cell death resulting from this accumulation of harmful disulfide bonds in the cytoplasm, triggering disulfide stress, is distinct from traditional regulated cell death pathways and represents a novel sulfur-dependent mode of cell death. Therefore, research into cell death mediated by disulfide bonds offers valuable insights for the development of innovative anticancer strategies.^[26]^

Cell death is a crucial mechanism for maintaining tissue homeostasis and preventing tumor development.^[38]^ Recent findings suggest that lncRNAs regulate programmed cell death pathways such as ferroptosis,^[22]^ cuproptosis,^[39]^ pyroptosis,^[40]^ and apoptosis^[41]^ in BLCA. However, the prognostic potential of disulfidptosis-related lncRNAs in BLCA remains unexplored. This study utilized bioinformatics analysis to evaluate the predictive efficacy of DRLs in BLCA progression. The research identified lncRNAs correlated with the expression of core genes involved in disulfidptosis and developed a prognostic model comprising 8 DRLs. This prognostic signature demonstrated superior predictive accuracy compared to other clinical features, as evidenced by the C-index and ROC curves. The comprehensive risk score incorporated into the prognostic nomogram model showed enhanced predictive precision over conventional clinical indicators alone. Calibration curves indicated a close correlation between actual outcomes and nomogram predictions. PCA of the risk model validated its effectiveness in distinguishing between low- and high-risk patients, reinforcing the robust prognostic value of the disulfide death lncRNA model for patients with BLCA. Furthermore, signatures of disulfidptosis-related lncRNAs may serve as predictive biomarkers across various cancer types. Notably, AC006160.1 is associated with ferroptosis and may act as a protective factor in BLCA progression.^[42]^ MIR4435-2HG is overexpressed in multiple tumor types, correlating with tumor initiation, progression, and poor prognosis.^[43]^ SMARCA5-AS1, LINC00513, and AL590428.1 have been utilized to construct predictive models for cuproptosis-related lncRNAs.^[44,45]^ Additionally, MIR4713HG has been shown to exacerbate the malignant behavior of oral squamous cell carcinoma by interacting with microRNA let-7c-5p.^[46]^ However, the molecular functions of AL359762.3 and AL122035.1 in various cancers remain to be elucidated.

Additionally, given the differential prognosis between high-risk and low-risk groups, enrichment analyses of GO, KEGG, and GSEA were conducted on DEGs in these groups. The DEGs primarily participate in key biological processes such as ECM organization, collagen-containing ECM, actin cytoskeleton dynamics in muscle cells, and the PI3K-AKT signaling pathway. ECM organization and collagen-containing ECM are crucial for tumor cell proliferation and invasion.^[47]^ The actin cytoskeleton of myocytes is associated with disulfide stress.^[48]^ Moreover, the PI3K pathway and ECM play pivotal roles in tumor immunity by enhancing immune cell activation and function and regulating tumor immune evasion.^[49,50]^

The tumor immune microenvironment is crucial in shaping disease progression and therapeutic responses in cancer.^[51]^ It has been shown that lncRNAs modulate tumor progression by influencing the TME.^[52]^ Immune infiltration analyses reveal a greater presence of M2 macrophages in the high-risk group, which are known for their immunosuppressive roles by secreting cytokines like IL-10 and TGF-β,^[53,54]^ potentially inhibiting T cell activation and proliferation. Additionally, plasma cells and B cells produce antibodies to identify and attack tumor cells consistent with better prognoses in the low-risk group have due to higher counts of these cells.^[55]^ Immune checkpoint inhibitors have revolutionized immunotherapy by reactivating the immune system’s ability to combat cancer.^[56]^ However, their effectiveness in BLCA is limited to certain patients, likely due to the absence of activated T lymphocytes at the tumor site.^[57]^ Analysis of immune functions associated with DRLs indicates increased immune checkpoint activity and suppression of antigen-presenting cells and T cell functions in the high-risk group, fostering an immunosuppressive environment that promotes immune evasion and disease progression. The relationship between TMB and immune checkpoints is currently a research focus.^[58]^ High TMB tumors generally have more mutations, generating a greater array of neoantigens that can elicit an immune response.^[59]^ Patients in the low-risk group tend to have higher TMB scores, potentially making them more responsive to immune checkpoint inhibitors. TIDE scores, used to assess immune evasion and suppression potential, show higher values in the high-risk group, indicating that these patients may benefit less from immunotherapy and are more prone to immune escape.^[60]^ Predictive modeling assessed BLCA’ sensitivity patients to various anticancer drugs. The analysis showed that high-risk patients are less responsive to 56 drug treatments compared to low-risk patients. Among these treatments, Ipatasertib, an AKT kinase inhibitor, induces mitochondrial apoptosis in BLCA cells by upregulating Bim,^[61]^ while ABT-737, a Bcl-2 family inhibitor, enhances apoptosis-inducing treatments by promoting necroptosis in urothelial cancer cells.^[62]^ Conventional chemotherapy agents such as cisplatin, paclitaxel, vinblastine, and docetaxel showed increased sensitivity in the high-risk group. Therefore, drug sensitivity assessments are crucial for tailoring therapeutic approaches in patients with BLCA.

In summary, research on DRLs holds significant potential for cancer prognosis prediction, guiding immunotherapy, elucidating tumor heterogeneity, drug development, and biomarker discovery. However, substantial knowledge gaps remain regarding the molecular mechanisms by which DRLs regulate genes associated with disulfidptosis. Bridging these gaps will necessitate integrating genomics, transcriptomics, and proteomics analyses, along with functional studies in cell and animal models. Over the next 5 years, it is anticipated that this field will yield deeper insights into the molecular mechanisms of DRLs, identify additional therapeutic targets, and bridge basic research with clinical data to develop novel diagnostic and therapeutic tools. Ultimately, these advancements will enhance personalized medicine and improve treatment outcomes for cancer patients. Additionally, it is important to acknowledge some limitations. Firstly, the results were validated only using the test set from TCGA, necessitating further validation with data from other databases to confirm the risk model’s reliability. Secondly, the bioinformatics predictions need to be validated through in vivo or in vitro models to explore the potential regulatory mechanisms of DRLs in BLCA. Finally, our DRL prognostic model requires validation with more samples obtained through prospective clinical trials to further ensure the model’s reliability.

## 5. Conclusions

This study represents the first attempt to construct a gene set of disulfidptosis-related lncRNAs associated with BLCA. By constructing this prognostic signature, a novel approach for predicting the prognosis of patients with BLCA is introduced. The model demonstrates significant associations with tumor immunity and offers potential guidance for treatment decisions in BLCA.

## Acknowledgments

We wish to extend our heartfelt thanks to the individuals who contributed to the publicly accessible databases that were instrumental in our research, as well as to the peer reviewers whose insightful feedback greatly enhanced our work.

## Author contributions

**Conceptualization:** Xiaoyu Yang, Yunzhi Zhang, Jun Liu, Yougang Feng.

**Data curation:** Xiaoyu Yang, Yunzhi Zhang, Jun Liu, Yougang Feng.

**Formal analysis:** Xiaoyu Yang, Yunzhi Zhang, Jun Liu, Yougang Feng.

**Software:** Xiaoyu Yang, Yunzhi Zhang.

**Supervision:** Jun Liu, Yougang Feng.

**Validation:** Yunzhi Zhang.

**Visualization:** Yunzhi Zhang, Jun Liu.

**Writing – original draft:** Xiaoyu Yang, Yunzhi Zhang.

**Writing – review & editing:** Yougang Feng.

## Supplementary Material

**Figure:**
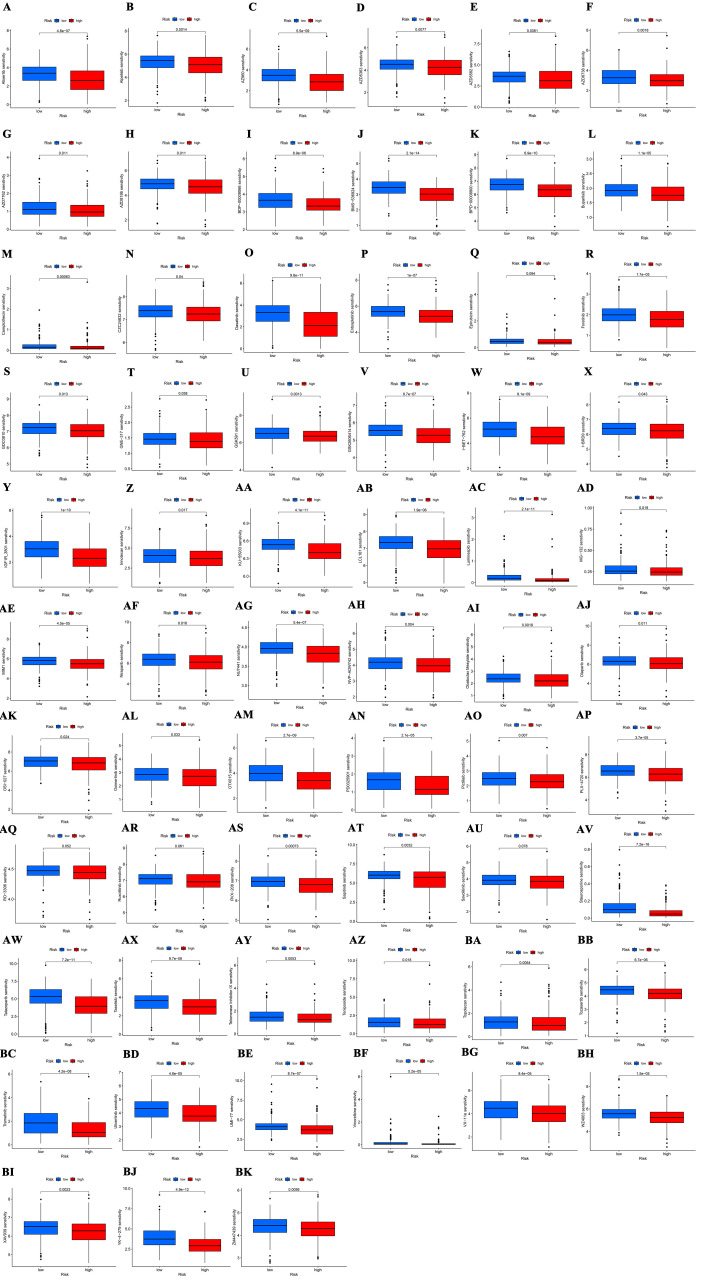


**Figure:**