# Supplementary material for: Construction and validation of a prognostic model for bladder cancer based on disulfidptosis-related lncRNAs
Source: Medicine (Baltimore). 2024 Jul 5;103(27):e38750. doi: 10.1097/MD.0000000000038750 (PMC11224815; doi:10.1097/MD.0000000000038750)
Supplement: Supplementary file 1 [file medi-103-e38750-s001.docx]

**Supplementary Table S1** IncRNAs associated with each of disulfidptosis gene in BLCA.

| Disulfidptosis | lncRNA | cor | pvalue | Regulation |
| --- | --- | --- | --- | --- |
| SLC7A11 | AC087286.4 | 0.429890763 | 7.95E-20 | postive |
| SLC7A11 | FAM155A-IT1 | 0.729983651 | 2.90E-69 | postive |
| SLC7A11 | RASA3-IT1 | 0.618078947 | 1.85E-44 | postive |
| SLC7A11 | ANKRD44-IT1 | 0.690327611 | 3.53E-59 | postive |
| NUBPL | AC073529.1 | 0.440050425 | 8.46E-21 | postive |
| SLC7A11 | AC138393.3 | 0.431788709 | 5.26E-20 | postive |
| SLC7A11 | MSC-AS1 | 0.465557902 | 2.16E-23 | postive |
| SLC7A11 | AL133371.2 | 0.51394123 | 5.98E-29 | postive |
| SLC7A11 | AL353593.1 | 0.553743375 | 3.07E-34 | postive |
| NDUFA11 | GATA3-AS1 | 0.506210712 | 5.31E-28 | postive |
| NDUFA11 | AC145423.1 | 0.450086795 | 8.57E-22 | postive |
| SLC7A11 | AC009269.4 | 0.43165841 | 5.41E-20 | postive |
| SLC7A11 | AC069549.1 | 0.650569287 | 1.40E-50 | postive |
| NDUFA11 | LINC02804 | 0.418729245 | 8.56E-19 | postive |
| SLC7A11 | IPO9-AS1 | 0.405688241 | 1.23E-17 | postive |
| NDUFA11 | AL035461.2 | 0.403592701 | 1.87E-17 | postive |
| NDUFA11 | AC087289.2 | 0.436993756 | 1.67E-20 | postive |
| SLC7A11 | AL512656.1 | 0.434020521 | 3.23E-20 | postive |
| NDUFA11 | SNHG11 | 0.46075609 | 6.91E-23 | postive |
| SLC7A11 | AL596223.1 | 0.775965473 | 1.80E-83 | postive |
| SLC7A11 | AC025031.2 | 0.456976655 | 1.70E-22 | postive |
| SLC7A11 | MIR4435-2HG | 0.486222446 | 1.17E-25 | postive |
| NDUFA11 | AC015912.3 | 0.433050459 | 3.99E-20 | postive |
| SLC7A11 | AL157394.3 | 0.476020352 | 1.61E-24 | postive |
| NDUFA11 | AC022966.2 | 0.489945061 | 4.40E-26 | postive |
| NUBPL | CASC2 | 0.405910507 | 1.18E-17 | postive |
| SLC7A11 | C2-AS1 | 0.424356276 | 2.61E-19 | postive |
| NUBPL | AC022364.1 | 0.454693213 | 2.92E-22 | postive |
| NCKAP1 | AC022364.1 | 0.463015851 | 4.01E-23 | postive |
| SLC7A11 | AC012568.1 | 0.454311563 | 3.20E-22 | postive |
| SLC7A11 | AC093484.4 | 0.416385754 | 1.39E-18 | postive |
| SLC7A11 | AC008569.2 | 0.491268698 | 3.10E-26 | postive |
| NDUFA11 | AL158063.1 | 0.431153874 | 6.04E-20 | postive |
| SLC7A11 | AC118555.1 | 0.659648923 | 1.98E-52 | postive |
| NDUFA11 | AC010331.1 | 0.463511122 | 3.55E-23 | postive |
| SLC7A11 | AC080188.1 | 0.605451344 | 2.87E-42 | postive |
| SLC7A11 | AC108463.2 | 0.458100249 | 1.30E-22 | postive |
| NDUFA11 | AC074212.1 | 0.431567834 | 5.52E-20 | postive |
| SLC7A11 | GSN-AS1 | 0.504729314 | 8.02E-28 | postive |
| NDUFA11 | AC002398.1 | 0.442117611 | 5.31E-21 | postive |
| SLC7A11 | AL133476.1 | 0.65389666 | 2.98E-51 | postive |
| NDUFA11 | AL645940.1 | 0.46660668 | 1.67E-23 | postive |
| NDUFA11 | AL390719.2 | 0.540773154 | 1.94E-32 | postive |
| SLC7A11 | AL031600.2 | 0.42739837 | 1.36E-19 | postive |
| SLC7A11 | DUBR | 0.527420994 | 1.16E-30 | postive |
| NDUFA11 | AC020765.2 | 0.458843643 | 1.09E-22 | postive |
| SLC7A11 | AC100763.1 | 0.416168201 | 1.46E-18 | postive |
| NUBPL | AL031670.1 | 0.43305624 | 3.99E-20 | postive |
| SLC7A11 | AC008443.2 | 0.409343121 | 5.91E-18 | postive |
| SLC7A11 | AC093515.1 | 0.460827508 | 6.79E-23 | postive |
| SLC7A11 | AL590428.1 | 0.406627378 | 1.02E-17 | postive |
| NDUFA11 | TP53TG1 | 0.474930607 | 2.12E-24 | postive |
| SLC7A11 | AL162724.2 | 0.68393276 | 1.05E-57 | postive |
| SLC7A11 | MIR223HG | 0.511232388 | 1.29E-28 | postive |
| NDUFA11 | AC067852.2 | 0.521935724 | 5.88E-30 | postive |
| SLC7A11 | LINC01687 | 0.463583288 | 3.49E-23 | postive |
| SLC7A11 | GRK5-IT1 | 0.618843379 | 1.35E-44 | postive |
| SLC7A11 | AC104984.2 | 0.428169586 | 1.15E-19 | postive |
| SLC7A11 | STARD13-IT1 | 0.651362418 | 9.68E-51 | postive |
| NDUFA11 | AL513320.1 | 0.50150254 | 1.95E-27 | postive |
| SLC7A11 | Z82243.1 | 0.508754544 | 2.60E-28 | postive |
| NUBPL | PAXIP1-AS2 | 0.436294643 | 1.95E-20 | postive |
| NCKAP1 | PAXIP1-AS2 | 0.408582763 | 6.89E-18 | postive |
| SLC7A11 | AL121957.1 | 0.424838273 | 2.36E-19 | postive |
| SLC7A11 | LINC01697 | 0.565862828 | 5.39E-36 | postive |
| SLC7A11 | AL136320.1 | 0.621764853 | 4.06E-45 | postive |
| SLC7A11 | AC011933.3 | 0.406241476 | 1.10E-17 | postive |
| NDUFA11 | AC009148.1 | 0.440454364 | 7.72E-21 | postive |
| SLC7A11 | AL139424.3 | 0.588947706 | 1.51E-39 | postive |
| SLC7A11 | GNG12-AS1 | 0.662625089 | 4.75E-53 | postive |
| SLC7A11 | Z99289.1 | 0.491815689 | 2.68E-26 | postive |
| NDUFS1 | AC073254.1 | 0.470568758 | 6.30E-24 | postive |
| SLC7A11 | AGAP1-IT1 | 0.4704705 | 6.45E-24 | postive |
| NUBPL | AC120114.1 | 0.437036443 | 1.66E-20 | postive |
| OXSM | ENTPD3-AS1 | 0.411902552 | 3.51E-18 | postive |
| NUBPL | RAP2C-AS1 | 0.454380181 | 3.14E-22 | postive |
| SLC7A11 | OSMR-AS1 | 0.50744485 | 3.76E-28 | postive |
| SLC7A11 | AC092053.3 | 0.658727805 | 3.07E-52 | postive |
| NDUFA11 | AC008610.1 | 0.607200458 | 1.45E-42 | postive |
| NDUFA11 | AC009065.8 | 0.446341955 | 2.03E-21 | postive |
| SLC7A11 | AL138999.1 | 0.718418006 | 3.83E-66 | postive |
| SLC7A11 | AC138207.4 | 0.683395097 | 1.40E-57 | postive |
| SLC7A11 | AC069277.1 | 0.649081811 | 2.76E-50 | postive |
| SLC7A11 | NCK1-DT | 0.529632908 | 5.95E-31 | postive |
| SLC7A11 | AL031429.2 | 0.492515626 | 2.22E-26 | postive |
| SLC7A11 | C1QTNF7-AS1 | 0.716887872 | 9.64E-66 | postive |
| SLC7A11 | LINC02256 | 0.466043283 | 1.92E-23 | postive |
| SLC7A11 | AC058791.1 | 0.51308669 | 7.63E-29 | postive |
| OXSM | AC008763.1 | 0.409335679 | 5.91E-18 | postive |
| NUBPL | AC011815.1 | 0.403648416 | 1.85E-17 | postive |
| NDUFA11 | AC009065.4 | 0.643022079 | 4.30E-49 | postive |
| SLC7A11 | ITGA9-AS1 | 0.468729949 | 9.92E-24 | postive |
| NDUFA11 | SNHG25 | 0.609438742 | 5.99E-43 | postive |
| SLC7A11 | AC090559.1 | 0.437470519 | 1.50E-20 | postive |
| NDUFA11 | AC010487.1 | 0.541633707 | 1.48E-32 | postive |
| SLC7A11 | DLEU2 | 0.439681617 | 9.19E-21 | postive |
| SLC7A11 | AC008750.4 | 0.498589848 | 4.33E-27 | postive |
| SLC7A11 | AC016831.1 | 0.59576921 | 1.18E-40 | postive |
| NDUFA11 | AC103706.1 | 0.449073519 | 1.08E-21 | postive |
| NDUFA11 | AC008764.6 | 0.51641665 | 2.93E-29 | postive |
| SLC7A11 | AC138207.7 | 0.56259464 | 1.63E-35 | postive |
| NDUFA11 | AC067852.5 | 0.503691093 | 1.07E-27 | postive |
| SLC7A11 | AC092301.1 | 0.421965424 | 4.34E-19 | postive |
| SLC7A11 | AC104984.5 | 0.464894074 | 2.54E-23 | postive |
| SLC7A11 | AC092807.3 | 0.538971864 | 3.41E-32 | postive |
| SLC7A11 | AC093520.1 | 0.402685144 | 2.24E-17 | postive |
| NDUFS1 | SMARCA5-AS1 | 0.420479369 | 5.93E-19 | postive |
| LRPPRC | SMARCA5-AS1 | 0.411091135 | 4.14E-18 | postive |
| NCKAP1 | SMARCA5-AS1 | 0.407463077 | 8.63E-18 | postive |
| LRPPRC | EMSLR | 0.453017809 | 4.33E-22 | postive |
| SLC7A11 | RBM5-AS1 | 0.452632433 | 4.74E-22 | postive |
| SLC7A11 | AC138207.9 | 0.736397704 | 4.58E-71 | postive |
| SLC7A11 | AL133227.1 | 0.402650713 | 2.26E-17 | postive |
| NDUFA11 | AL008582.1 | 0.423899574 | 2.88E-19 | postive |
| SLC7A11 | AC087854.1 | 0.545415632 | 4.50E-33 | postive |
| SLC7A11 | AC011092.3 | 0.411469967 | 3.83E-18 | postive |
| NDUFA11 | AL021707.8 | 0.50030563 | 2.71E-27 | postive |
| SLC7A11 | AC011510.1 | 0.671109475 | 7.42E-55 | postive |
| SLC7A11 | AC024267.5 | 0.40357761 | 1.88E-17 | postive |
| NDUFA11 | HEIH | 0.431940233 | 5.09E-20 | postive |
| SLC7A11 | HMGA2-AS1 | 0.67427016 | 1.52E-55 | postive |
| NUBPL | AL035411.3 | 0.418508829 | 8.97E-19 | postive |
| NCKAP1 | AL035411.3 | 0.449522581 | 9.77E-22 | postive |
| SLC7A11 | AC087286.1 | 0.525865626 | 1.84E-30 | postive |
| SLC7A11 | AC110609.1 | 0.504546125 | 8.44E-28 | postive |
| SLC7A11 | AC012358.2 | 0.46806796 | 1.17E-23 | postive |
| NDUFA11 | AL096701.4 | 0.414423731 | 2.09E-18 | postive |
| SLC7A11 | RNF216-IT1 | 0.482340748 | 3.21E-25 | postive |
| SLC7A11 | AL356234.3 | 0.578272174 | 7.19E-38 | postive |
| SLC7A11 | AC009716.1 | 0.61469675 | 7.31E-44 | postive |
| SLC7A11 | AC012076.1 | 0.455942662 | 2.18E-22 | postive |
| SLC7A11 | AL139042.1 | 0.608919141 | 7.35E-43 | postive |
| SLC7A11 | LINC00536 | 0.71426222 | 4.64E-65 | postive |
| SLC7A11 | JARID2-AS1 | 0.478436525 | 8.72E-25 | postive |
| SLC7A11 | AL445649.1 | 0.583371439 | 1.16E-38 | postive |
| SLC7A11 | AL137779.1 | 0.491802769 | 2.69E-26 | postive |
| NDUFA11 | MHENCR | 0.548133902 | 1.89E-33 | postive |
| SLC7A11 | AC010525.1 | 0.452514354 | 4.87E-22 | postive |
| SLC7A11 | RBMS3-AS2 | 0.705118091 | 9.62E-63 | postive |
| NDUFA11 | AC016773.1 | 0.438022329 | 1.33E-20 | postive |
| NDUFA11 | AC023302.1 | 0.468666942 | 1.01E-23 | postive |
| NDUFA11 | AL355353.1 | 0.522480986 | 5.01E-30 | postive |
| NDUFA11 | AC124016.1 | 0.430342522 | 7.21E-20 | postive |
| SLC7A11 | LAMC1-AS1 | 0.487295163 | 8.84E-26 | postive |
| NDUFA11 | AC027682.4 | 0.431122825 | 6.08E-20 | postive |
| SLC7A11 | AL353804.2 | 0.457289522 | 1.58E-22 | postive |
| NDUFA11 | AL139123.1 | 0.523427473 | 3.79E-30 | postive |
| SLC7A11 | AC079921.1 | 0.703304642 | 2.70E-62 | postive |
| SLC7A11 | AC066613.1 | 0.503419512 | 1.15E-27 | postive |
| SLC7A11 | AC104472.5 | 0.470989215 | 5.67E-24 | postive |
| SLC7A11 | AC020978.2 | 0.512870972 | 8.11E-29 | postive |
| SLC7A11 | AC079921.2 | 0.621203634 | 5.12E-45 | postive |
| NUBPL | AL353804.1 | 0.404350255 | 1.61E-17 | postive |
| SLC7A11 | AL353804.1 | 0.49898168 | 3.89E-27 | postive |
| SLC7A11 | SAP30L-AS1 | 0.502926027 | 1.32E-27 | postive |
| NDUFS1 | AC015922.2 | 0.406056196 | 1.14E-17 | postive |
| NCKAP1 | AC015922.2 | 0.414381998 | 2.11E-18 | postive |
| NDUFA11 | AL163051.1 | 0.443820045 | 3.61E-21 | postive |
| NDUFA11 | AP003419.3 | 0.562635989 | 1.61E-35 | postive |
| NUBPL | AC108449.2 | 0.438447127 | 1.21E-20 | postive |
| SLC7A11 | AC108449.2 | 0.412986468 | 2.81E-18 | postive |
| NDUFA11 | SBNO1-AS1 | 0.403055782 | 2.08E-17 | postive |
| SLC7A11 | AL355102.4 | 0.527998326 | 9.73E-31 | postive |
| NUBPL | AC018752.1 | 0.422836878 | 3.61E-19 | postive |
| SLC7A11 | AC018752.1 | 0.459080275 | 1.03E-22 | postive |
| SLC7A11 | AC083870.1 | 0.560806227 | 2.97E-35 | postive |
| SLC7A11 | AC215522.2 | 0.531168032 | 3.74E-31 | postive |
| NDUFA11 | AC073508.3 | 0.423556795 | 3.10E-19 | postive |
| SLC7A11 | AC009268.2 | 0.402822454 | 2.18E-17 | postive |
| SLC7A11 | LRIG2-DT | 0.657152011 | 6.47E-52 | postive |
| NDUFA11 | AL023284.4 | 0.46972157 | 7.77E-24 | postive |
| SLC7A11 | MIR222HG | 0.479879225 | 6.03E-25 | postive |
| NDUFA11 | LINC01089 | 0.540820673 | 1.92E-32 | postive |
| SLC7A11 | LSAMP-AS1 | 0.662415524 | 5.26E-53 | postive |
| SLC7A11 | STARD13-AS | 0.672970608 | 2.92E-55 | postive |
| NDUFA11 | AC011445.1 | 0.435718821 | 2.22E-20 | postive |
| NUBPL | AL139353.2 | 0.407540557 | 8.50E-18 | postive |
| NDUFA11 | AC011462.4 | 0.482941578 | 2.75E-25 | postive |
| SLC7A11 | AC007922.2 | 0.460524905 | 7.30E-23 | postive |
| NDUFA11 | AC008915.2 | 0.459427491 | 9.50E-23 | postive |
| SLC7A11 | LINC02577 | 0.456293033 | 2.00E-22 | postive |
| SLC7A11 | SH3RF3-AS1 | 0.457969512 | 1.35E-22 | postive |
| SLC7A11 | AL512603.2 | 0.448732754 | 1.17E-21 | postive |
| NCKAP1 | C2orf27A | 0.486981766 | 9.60E-26 | postive |
| SLC7A11 | AC005730.3 | 0.459858162 | 8.57E-23 | postive |
| SLC7A11 | AC087286.2 | 0.475469347 | 1.85E-24 | postive |
| SLC7A11 | AC037487.2 | 0.575349557 | 2.02E-37 | postive |
| SLC7A11 | AC005740.4 | 0.516246819 | 3.08E-29 | postive |
| NDUFS1 | AL132800.1 | 0.412897365 | 2.86E-18 | postive |
| NUBPL | AL132800.1 | 0.493732215 | 1.61E-26 | postive |
| NDUFA11 | AC127024.6 | 0.448197868 | 1.33E-21 | postive |
| NUBPL | AC009041.3 | 0.413071213 | 2.76E-18 | postive |
| SLC7A11 | AC007620.3 | 0.524447214 | 2.80E-30 | postive |
| LRPPRC | SNHG29 | 0.413806307 | 2.38E-18 | postive |
| NDUFA11 | ARHGAP27P1-BPTFP1-KPNA2P3 | 0.411520398 | 3.79E-18 | postive |
| SLC7A11 | AL137186.1 | 0.421770037 | 4.52E-19 | postive |
| SLC7A11 | SGMS1-AS1 | 0.478571272 | 8.42E-25 | postive |
| SLC7A11 | IQCJ-SCHIP1-AS1 | 0.64422785 | 2.50E-49 | postive |
| SLC7A11 | AL138995.1 | 0.408609063 | 6.85E-18 | postive |
| NDUFA11 | AC046143.2 | 0.405274737 | 1.34E-17 | postive |
| SLC7A11 | LINC02649 | 0.438933186 | 1.09E-20 | postive |
| NDUFA11 | SNHG12 | 0.465307806 | 2.30E-23 | postive |
| SLC7A11 | AC090948.1 | 0.401821701 | 2.66E-17 | postive |
| NDUFA11 | MIR200CHG | 0.491236962 | 3.13E-26 | postive |
| NDUFS1 | AC107027.3 | 0.420580281 | 5.81E-19 | postive |
| SLC7A11 | RERG-IT1 | 0.629605725 | 1.51E-46 | postive |
| NUBPL | AL157400.4 | 0.414414207 | 2.10E-18 | postive |
| SLC7A11 | KLF7-IT1 | 0.566224416 | 4.76E-36 | postive |
| SLC7A11 | AC104170.1 | 0.480886655 | 4.66E-25 | postive |
| SLC7A11 | LINC01203 | 0.508922884 | 2.48E-28 | postive |
| SLC7A11 | AP000829.1 | 0.620010583 | 8.38E-45 | postive |
| NDUFA11 | AC132872.2 | 0.427327627 | 1.38E-19 | postive |
| NCKAP1 | OGFRP1 | 0.421494224 | 4.79E-19 | postive |
| SLC7A11 | AL359762.3 | 0.415109821 | 1.82E-18 | postive |
| SLC7A11 | AC130371.1 | 0.66208813 | 6.15E-53 | postive |
| NDUFA11 | AL035587.2 | 0.525290059 | 2.18E-30 | postive |
| NCKAP1 | AC097448.1 | 0.404706513 | 1.50E-17 | postive |
| NUBPL | LINC01184 | 0.448691417 | 1.18E-21 | postive |
| NDUFA11 | AC024060.2 | 0.557497245 | 8.94E-35 | postive |
| RPN1 | AC137695.3 | 0.455184941 | 2.60E-22 | postive |
| SLC7A11 | AC092171.1 | 0.450869953 | 7.15E-22 | postive |
| NDUFA11 | AC010422.2 | 0.554462753 | 2.43E-34 | postive |
| SLC7A11 | LRRC8C-DT | 0.414861923 | 1.91E-18 | postive |
| NDUFA11 | AC010618.2 | 0.555586662 | 1.68E-34 | postive |
| SLC7A11 | MAP3K20-AS1 | 0.615616239 | 5.04E-44 | postive |
| SLC7A11 | AC083798.2 | 0.417346304 | 1.14E-18 | postive |
| SLC7A11 | AC108053.1 | 0.46059872 | 7.18E-23 | postive |
| NDUFA11 | SNHG9 | 0.470406768 | 6.55E-24 | postive |
| NDUFA11 | AC098484.2 | 0.40559144 | 1.26E-17 | postive |
| SLC7A11 | LINC01484 | 0.577877264 | 8.27E-38 | postive |
| SLC7A11 | AC074194.1 | 0.675073884 | 1.01E-55 | postive |
| SLC7A11 | TCF4-AS1 | 0.689435033 | 5.70E-59 | postive |
| SLC7A11 | FENDRR | 0.532067231 | 2.85E-31 | postive |
| NDUFA11 | RAB11B-AS1 | 0.580049885 | 3.82E-38 | postive |
| NUBPL | CKMT2-AS1 | 0.425037779 | 2.26E-19 | postive |
| SLC7A11 | AP003171.1 | 0.643792555 | 3.04E-49 | postive |
| NUBPL | AL391840.3 | 0.412524621 | 3.09E-18 | postive |
| NDUFA11 | AC007292.1 | 0.538504906 | 3.94E-32 | postive |
| NDUFA11 | AC005387.1 | 0.602006818 | 1.09E-41 | postive |
| NDUFA11 | AC138230.1 | 0.440421233 | 7.78E-21 | postive |
| SLC7A11 | AC090907.3 | 0.527456777 | 1.14E-30 | postive |
| SLC7A11 | LINC02595 | 0.598378173 | 4.40E-41 | postive |
| SLC7A11 | AL133445.2 | 0.603420821 | 6.33E-42 | postive |
| NDUFA11 | AL021707.6 | 0.544959832 | 5.20E-33 | postive |
| NDUFA11 | ZFAS1 | 0.499905084 | 3.03E-27 | postive |
| SLC7A11 | AP003900.1 | 0.743217591 | 4.86E-73 | postive |
| NDUFA11 | AL121832.2 | 0.427388469 | 1.37E-19 | postive |
| NDUFA11 | KMT2E-AS1 | 0.499966752 | 2.98E-27 | postive |
| SLC7A11 | AL031651.2 | 0.641386059 | 8.92E-49 | postive |
| SLC7A11 | AC025280.1 | 0.44464542 | 2.99E-21 | postive |
| NDUFA11 | AC008608.2 | 0.426092329 | 1.80E-19 | postive |
| SLC7A11 | Z69666.1 | 0.501597087 | 1.90E-27 | postive |
| SLC7A11 | AC009054.1 | 0.433936459 | 3.29E-20 | postive |
| NDUFS1 | AL356019.2 | 0.408615293 | 6.84E-18 | postive |
| NUBPL | AL356019.2 | 0.49919324 | 3.68E-27 | postive |
| NCKAP1 | AL356019.2 | 0.425844435 | 1.90E-19 | postive |
| SLC7A11 | AC107308.1 | 0.450855314 | 7.17E-22 | postive |
| SLC7A11 | LINC00513 | 0.566326687 | 4.60E-36 | postive |
| SLC7A11 | AC104564.5 | 0.430058616 | 7.67E-20 | postive |
| SLC7A11 | LINC00308 | 0.628168417 | 2.78E-46 | postive |
| NDUFS1 | AC024075.1 | 0.41964599 | 7.07E-19 | postive |
| SLC7A11 | SLC7A11-AS1 | 0.757875388 | 1.67E-77 | postive |
| NDUFA11 | AL354836.1 | 0.462270698 | 4.80E-23 | postive |
| OXSM | AL139287.1 | 0.40341609 | 1.94E-17 | postive |
| SLC7A11 | AC067852.3 | 0.463134224 | 3.89E-23 | postive |
| SLC7A11 | NXT1-AS1 | 0.52071663 | 8.41E-30 | postive |
| SLC7A11 | AC099811.5 | 0.446785544 | 1.84E-21 | postive |
| NDUFA11 | H1-10-AS1 | 0.554271135 | 2.58E-34 | postive |
| NDUFA11 | AC120053.1 | 0.441820702 | 5.68E-21 | postive |
| SLC7A11 | AC025280.3 | 0.475141516 | 2.01E-24 | postive |
| SLC7A11 | AL133243.3 | 0.400866597 | 3.21E-17 | postive |
| SLC7A11 | FO680682.1 | 0.471863386 | 4.56E-24 | postive |
| SLC7A11 | AC007731.3 | 0.403929251 | 1.75E-17 | postive |
| LRPPRC | AL161891.1 | 0.408793483 | 6.60E-18 | postive |
| SLC7A11 | AL161891.1 | 0.428589151 | 1.05E-19 | postive |
| SLC7A11 | AC004846.2 | 0.656179657 | 1.02E-51 | postive |
| SLC7A11 | AC019183.1 | 0.533172456 | 2.03E-31 | postive |
| SLC7A11 | DST-AS1 | 0.506884425 | 4.40E-28 | postive |
| SLC7A11 | AC023825.2 | 0.665547031 | 1.15E-53 | postive |
| SLC7A11 | AL355102.1 | 0.724340617 | 1.01E-67 | postive |
| NUBPL | AC004943.2 | 0.437230448 | 1.59E-20 | postive |
| SLC7A11 | AC073569.3 | 0.441588396 | 5.98E-21 | postive |
| SLC7A11 | AC100830.1 | 0.444683957 | 2.97E-21 | postive |
| SLC7A11 | AC022154.1 | 0.453490271 | 3.88E-22 | postive |
| SLC7A11 | AC019080.5 | 0.40956354 | 5.65E-18 | postive |
| SLC7A11 | AC090673.1 | 0.627224781 | 4.14E-46 | postive |
| SLC7A11 | WWTR1-IT1 | 0.713284039 | 8.29E-65 | postive |
| NDUFA11 | Z69706.1 | 0.410281426 | 4.88E-18 | postive |
| OXSM | AC112220.2 | 0.514665329 | 4.86E-29 | postive |
| NDUFA11 | AC087741.1 | 0.431867568 | 5.17E-20 | postive |
| SLC7A11 | AC004832.4 | 0.474377021 | 2.43E-24 | postive |
| SLC7A11 | NEXN-AS1 | 0.515618436 | 3.69E-29 | postive |
| NDUFA11 | AL590101.1 | 0.404713113 | 1.50E-17 | postive |
| SLC7A11 | SALRNA1 | 0.582144467 | 1.80E-38 | postive |
| NDUFS1 | AC005670.3 | 0.458461503 | 1.20E-22 | postive |
| NUBPL | AC005670.3 | 0.430084499 | 7.63E-20 | postive |
| SLC7A11 | AL355073.2 | 0.462867781 | 4.15E-23 | postive |
| SLC7A11 | AC006160.1 | 0.587015906 | 3.07E-39 | postive |
| SLC7A11 | AC063944.2 | 0.464323743 | 2.92E-23 | postive |
| SLC7A11 | AL138759.1 | 0.606554947 | 1.87E-42 | postive |
| NDUFA11 | AC145285.6 | 0.457583023 | 1.47E-22 | postive |
| SLC7A11 | AC108463.3 | 0.549329313 | 1.29E-33 | postive |
| NDUFA11 | AC104785.1 | 0.42987395 | 7.98E-20 | postive |
| SLC7A11 | AC146507.3 | 0.400694286 | 3.32E-17 | postive |
| SLC7A11 | AC007319.1 | 0.474122236 | 2.59E-24 | postive |
| NDUFA11 | RPARP-AS1 | 0.514883076 | 4.56E-29 | postive |
| SLC7A11 | KIF26B-AS1 | 0.465517928 | 2.18E-23 | postive |
| SLC7A11 | AC093620.1 | 0.413973099 | 2.30E-18 | postive |
| SLC7A11 | AC100810.3 | 0.535465929 | 1.01E-31 | postive |
| SLC7A11 | AC021205.3 | 0.702008604 | 5.63E-62 | postive |
| SLC7A11 | LINC-PINT | 0.464855374 | 2.56E-23 | postive |
| SLC7A11 | Z98885.3 | 0.412544744 | 3.08E-18 | postive |
| NDUFA11 | AL691432.4 | 0.459585567 | 9.15E-23 | postive |
| SLC7A11 | FAM230C | 0.480758782 | 4.82E-25 | postive |
| NDUFA11 | AC090425.2 | 0.470465462 | 6.46E-24 | postive |
| SLC7A11 | ZFPM2-AS1 | 0.404731706 | 1.49E-17 | postive |
| SLC7A11 | AC080023.1 | 0.463407247 | 3.64E-23 | postive |
| NDUFA11 | AC112484.3 | 0.405283206 | 1.34E-17 | postive |
| SLC7A11 | AC040904.1 | 0.62472488 | 1.18E-45 | postive |
| NCKAP1 | MIR4713HG | 0.44404767 | 3.43E-21 | postive |
| SLC7A11 | PART1 | 0.518438447 | 1.63E-29 | postive |
| SLC7A11 | AC234772.1 | 0.679412791 | 1.10E-56 | postive |
| RPN1 | AL441992.1 | 0.446880248 | 1.80E-21 | postive |
| SLC7A11 | AC006159.1 | 0.6167068 | 3.24E-44 | postive |
| SLC7A11 | AL731537.1 | 0.714435647 | 4.18E-65 | postive |
| NUBPL | AL122035.1 | 0.401030436 | 3.11E-17 | postive |
